# Ursolic Acid Inhibits Leucine-Stimulated mTORC1 Signaling by Suppressing mTOR Localization to Lysosome

**DOI:** 10.1371/journal.pone.0095393

**Published:** 2014-04-16

**Authors:** Xiang Ou, Meilian Liu, Hairong Luo, Lily Q. Dong, Feng Liu

**Affiliations:** 1 Metabolic Syndrome Research Center and Diabetes Center, Key Laboratory of Diabetes Immunology, Second Xiangya Hospital, Central South University, Changsha, Hunan, China; 2 Department of Pharmacology University of Texas Health Science Center at San Antonio, San Antonio, Texas, United States of America; 3 Department of Cellular & Structural Biology, University of Texas Health Science Center at San Antonio, San Antonio, Texas, United States of America; Boston University School of Medicine, United States of America

## Abstract

Ursolic acid (UA), a pentacyclic triterpenoid widely found in medicinal herbs and fruits, has been reported to possess a wide range of beneficial properties including anti-hyperglycemia, anti-obesity, and anti-cancer. However, the molecular mechanisms underlying the action of UA remain largely unknown. Here we show that UA inhibits leucine-induced activation of the mechanistic target of rapamycin complex 1 (mTORC1) signaling pathway in C2C12 myotubes. The UA-mediated inhibition of mTORC1 is independent of Akt, tuberous sclerosis complex 1/2 (TSC1/2), and Ras homolog enriched in brain (Rheb), suggesting that UA negatively regulates mTORC1 signaling by targeting at a site downstream of these mTOR regulators. UA treatment had no effect on the interaction between mTOR and its activator Raptor or inhibitor Deptor, but suppressed the binding of RagB to Raptor and inhibited leucine-induced mTOR lysosomal localization. Taken together, our study identifies UA as a direct negative regulator of the mTORC1 signaling pathway and suggests a novel mechanism by which UA exerts its beneficial function.

## Introduction

Ursolic acid (UA) is a triterpene compound derived from certain traditional medicinal plants [1]. UA has been found to possess a wide range of health benefits such as anti-obesity-related diseases [2], anti-hyperglycemia [3] and anti-cancer [4]. However, the molecular mechanisms underlying the beneficial effects of UA remain largely unknown. UA has been shown to ameliorate hepatic fibrosis by decreasing Akt phosphorylation and blocking NFκB nuclear localization in mouse hepatocytes [5]. Very recently, UA has also been found to inhibit the initiation and progression of prostate cancer by down-regulation of various pro-inflammatory mediators including NF-κB, STAT3, Akt and IKKα/β [6]. However, whether UA has additional targets remains to be determined.

The mechanistic target of rapamycin (mTOR) is a serine-threonine kinase that plays important roles in the regulation of protein and lipid synthesis, autophagy, as well as mitochondrial metabolism and biogenesis [7], [8]. mTOR interacts with several proteins to form two distinct multiprotein complexes named mTORC1 and mTORC2 [9]. While mTOR, Deptor, and mLST8 are present in both complexes, Raptor and Rictor are present in mTORC1 and mTORC2, respectively. Among these components, Raptor and Deptor positively and negatively regulate mTOR activation, respectively [10], [11]. mTORC1 signaling is regulated by a variety of upstream signals including growth factors, amino acids, energy status and oxygen concentration. Insulin regulates mTORC1 activity by activation of the class I PI3K/Akt signaling pathway [9]. Amino acids, on the other hand, have been shown to activate mTORC1 by signaling through the class 3 PI3K, hVps34 [12]. Insulin-stimulated activation of the PI3K/Akt signaling pathway leads to the inhibition of TSC1/TSC2, resulting in activation of small GTPase Rheb and subsequent activation of mTORC1 [13], [14], [15]. The mechanisms by which mTORC1 is activated by amino acids are less clear but a number of studies have shown that the binding of Raptor to the Rag GTPases, a family of four related small GTPases [16], is necessary for mTOR to localize onto the lysosome where mTOR is activated by its upstream activator Rheb [16], [17], .

While the mTORC1 signaling pathway has been well established as a key component to promote growth and accelerate cancer, it also plays an important role in regulating metabolism [20], [21], [22]. Skeletal muscle is the major site of glucose disposal and impairment in muscle glucose uptake contributes to type 2 diabetes [23]. Diet-induced over-activation of mTORC1 in muscle has been shown to inhibit insulin signaling via a feedback mechanism involving S6K-mediated serine phosphorylation of IRS-1, which reduces the ability of IRS1 to activate PI3K, leading to impaired glucose uptake and systemic insulin resistance [24]. Preventing over-activation of the mTORC1 signaling pathway in muscle could thus provide an effective treatment for obesity-induced insulin resistance and metabolic dysfunction.

In the current study, we investigated the effect of UA on mTOR signaling, we found that UA inhibits leucine-stimulated mTOR activation in C2C12 myotubes by inhibiting mTOR from targeting to lysosome, uncovering a novel mechanism underlying the negative regulation of the mTORC1 signaling pathway by UA, a small molecule that possess a wide range of beneficial properties including anti-hyperglycemia, anti-obesity, and anti-cancer.

## Materials and Methods

### Plasmids and Reagents

Plasmids encoding FLAG-tagged wild-type and Q99L mutant of RagB, the lentiviral vector pWPI, the lentiviral envelope plasmids pMD2.G and psPAX2 were obtained from Addgene. The plasmids encoding wild-type and S16H mutant of Rheb were gifts of Dr. Bo Xiao (Johns Hopkins University School of Medicine) and were described previously [25]. Leucine, UA, the FLAG M2 antibody, and Cocktail 3 were from Sigma Aldrich. The Anti-LAMP1 antibody was from ABCAM. Alexa Fluor 488 Donkey anti-Mouse IgG (H+L) antibody and Alexa Fluor 568 Donkey Anti-Rabbit IgG antibody were from Invitrogen. Protein A-Sepharose beads were from GE Healthcare Life Sciences. Clarity Western ECL substrate was from bio-rad. HRP labeled anti-mouse and anti-rabbit secondary antibodies were from Promega. Normal rabbit IgG and normal mouse IgG were from Millipore; the RagB antibody was from Proteintech. All other antibodies were from Cell Signaling Technology.

### Cell Culture

C2C12 cells (American Type Culture Collection) were cultured in DMEM (ATCC 30-2002) supplemented with 10% newborn calf serum and 1% penicillin/streptomycin. TSC1/2^−/−^ and wild-type mouse embryonic fibroblasts (MEFs) (generous gifts of Dr. Kunliang Guan, University of California at San Diego) were grown in DMEM (Gibco 11995-065) supplemented with 10% new born calf serum and 1% penicillin/streptomycin [26]. PDK1 Knock-out MEF cells, Deptor-suppressed C2C12 cells and their wild-type control cells were described previously [26].

### Generation of C2C12 Cells Expressing RagB^wt^, RagB^Q99L^ Mutant, Rheb^wt^, Or Rheb^S16H^ Mutant

cDNAs encoding wild-type RagB, Q99L mutant of RagB, wild-type Rheb, S16H mutant of Rheb were subcloned into the lentiviral vector pWPI. Lentiviral particles were produced by transfecting 293T cells with the pMD2.G plasmid, the psPAX2 plasmid, and the pWPI plasmid encoding green fluorescent protein (GFP) vector, RagB^wt^, RagB^Q99L^_,_ Rheb^wt^, or Rheb^S16H^. The medium was changed to growth medium eight hours post transfection and collected after 48 hours. The collected medium was filtered via a 0.45-µm filter and stored at −80°C until needed. For cell infection, C2C12 cells were incubated with the lentiviral medium in the presence of 8 µg/ml polybrene for 72 hours. Cells were visually scored for GFP intensity. Control cells were infected with lentiviruses encoding only GFP. No notable difference in proliferation and behave was detected between infected and uninfected parental cells.

### Western Blot and Immunoprecipitation (IP)

Cells were rinsed once with ice-cold PBS and lysed in ice-cold lysis buffer (40 mM HEPES [pH 7.4], 2 mM EDTA, 10 mM pyrophosphate, 10 mM glycerophosphate, and 0.3% CHAPS, 1 mM sodium orthovanadate, 10 ug/ml leupeptin, 10 ug/ml aprotinin, 1 mM phenylmethanesulfonyl fluoride, and phosphatase inhibitor mixtures). Cell lysates were centrifuged at 13,000 rpm for 10 minutes and supernatants were collected. For IP experiments, primary antibodies were added to the lysates and incubated with rotation overnight at 4°C. After addition of 30 µl of 50% slurry of the protein A-sepharose, the incubation was continued for 2 more hours. Immunoprecipitates were washed three times with ice-cold lysis buffer containing 150 mM NaCl. Immunoprecipitated proteins were detected by Western blot using 8%–12% SDS-PAGE as described [27].

### In-cell Cross-linking Reaction

The in-cell cross-linking experiments were performed according to the procedure described previously [16]. In brief, DSP (Dithiobis[succinimidyl propionate]) was dissolved in DMSO (Dimethyl sulfoxide) to a final concentration of 250 mg/ml to make a 250X stock solution for the in-cell cross-linking assay. C2C12 cells transiently expressing FLAG-tagged RagB grown on 100-mm plates were serum starved and pretreated with or without 50 µM UA for 60 minutes, followed with or without 10 mM leucine for 60 minutes. At the end of the treatment, cells were rinsed once with cold PBS and incubated with 4 ml of PBS containing 1 mg/ml DSP for 7 minutes at room temperature. The cross-linking reaction was quenched by the addition of 1 M Tris buffer (pH 8.5) to a final concentration of 100 mM. After one-minute incubation, the cells were rinsed once with ice-cold PBS and lysed with lysis buffer containing 40 mM HEPES (pH 7.4), 2 mM EDTA, 10 mM pyrophosphate, 10 mM glycerophosphate, 0.3% CHAPS, 1 mM sodium orthovanadate, 10 ug/ml leupeptin, 10 ug/ml aprotinin, 1 mM phenylmethanesulfonyl fluoride, and phosphatase inhibitor mixtures. Immunoprecipitations using antibodies to Raptor or the FLAG tag were performed as described above.

### Immunofluorescence Assays

Cells were grown on round cover slips in 12-well plates to 60–80% confluence. Cells were serum starved, treated with or without leucine and/or UA, rinsed with PBS, and fixed with 4% paraformaldehyde for 5 minutes at room temperature. The coverslips were rinsed twice with PBS and permealized with 0.2% Triton X-100 in PBS for 15 minutes. After rinsing two more times with PBS, the coverslips were blocked for one hour in blocking buffer (0.25% BSA in PBS) and incubated with primary antibody overnight at 4°C. After rinsing with blocking buffer, cells were incubated with secondary antibodies for one hour at room temperature. The coverslips were mounted on glass slides using glycerol and the fluorescence images were detected by a Zeiss LSM780 NLO fluorescence microscope with a 63 x objective.

### Statistical Analysis

Quantification of protein phosphorylation and total levels was performed by analyzing Western blots using the NIH Image J program. Statistical analysis of the data was performed using the analysis of variance (ANOVA). Data were presented as mean ± S.E.M. Statistical significance was set at *P* values of *<0.05, **<0.01, and ***<0.001.

## Results

### UA Inhibits Leucine-stimulated mTORC1 Signaling via a PI3K/Akt-independent Mechanism

**Figure 1:**
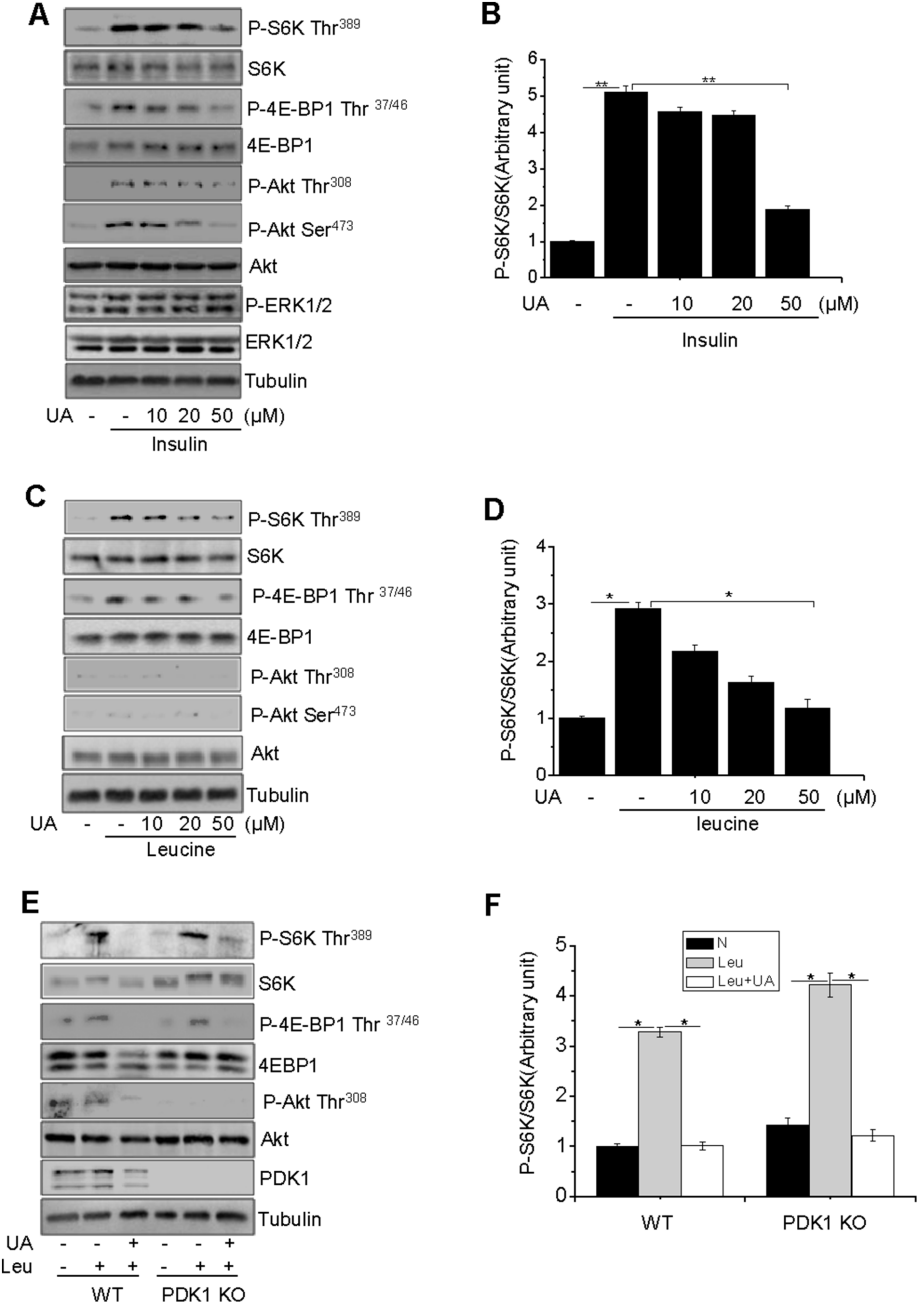
UA inhibits leucine-stimulated mTOR activation via a PI3K–Akt-independent manner. (**A**), Serum-starved C2C12 myotubes were pretreated with or without UA at the indicated concentrations for 60 min, followed with or without 10 nM insulin (Ins) for 10 min (**A**) or 10 mM leucine (Leu) for 60 min (**C**). The phosphorylation and protein levels of Akt, S6K, 4EBP1, and ERK1/2 were determined by Western blot with indicated antibodies. (**B**) and (**D**), S6K phosphorylation in (**A**) and (**C**) was semi-quantified using the NIH Image J Program and normalized to S6K protein levels. (**E**), Serum-starved wild type and PDK1-knockout (KO) MEFs were pretreated with or without 50 µM UA for 60 minutes, followed with or without 10 mM leucine (Leu) for 60 minutes. The phosphorylation and protein levels of the interesting signaling molecules were determined by Western blot using specific antibodies as indicated. (**F**), S6K phosphorylation in (**E**) was semi-quantified using the NIH Image J Program and normalized to S6K protein levels. Tubulin was used as a loading control for all experiments, differences between groups were examined for statistical significance using ANOVA. Data are presented as mean±S.E.M. from three independent experiments. *, *P*<0.05, **P<0.01, N, no addition.

To determine the potential effect of UA on mTOR activity, differentiated C2C12 myotubes were treated with UA at different doses, followed with insulin or leucine treatment. Insulin treatment greatly stimulated the phosphorylation of S6K, 4EBP1, and Akt, and this phosphorylation was suppressed by UA treatment in a dose-dependent manner (Figs 1A and 1B). Insulin also stimulated the phosphorylation of ERK1/2, but this phosphorylation was not inhibited by UA (Fig. 1A). Unlike insulin, leucine stimulated the phosphorylation of S6K and 4EBP1, but had little effect on Akt phosphorylation (Figs. 1C and 1D), which is consistent with the finding that leucine had no effect on Akt phosphorylation in skeletal muscle cells [28], [29], [30], [31]. In addition, UA inhibited leucine-stimulated S6K phosphorylation in PDK1 knockout MEFs (Figs. 1 E and 1F). Pretreatment of C2C12 myotubes with UA had little effect on the protein levels of Raptor, Rictor, and mTOR (data not shown). Taken together, these results suggest that the UA is able to inhibit mTORC1 signaling via a novel mechanism.

### UA Inhibits Leucine-stimulated, mTOR Activation in a TSC1/2 and Rheb-independent Manner

**Figure 2:**
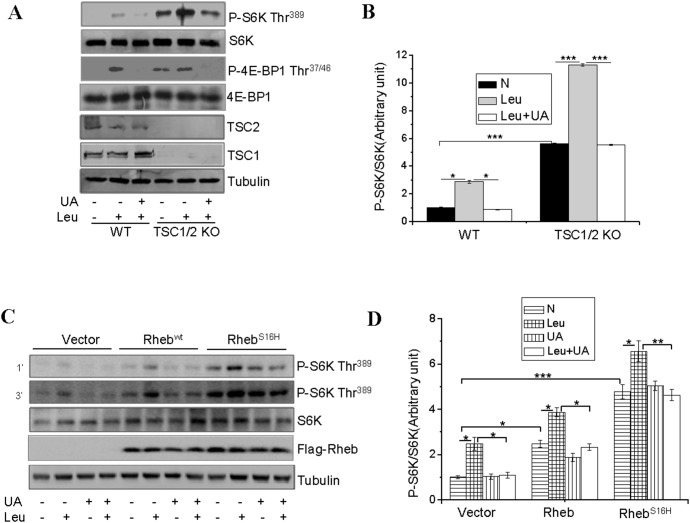
UA inhibits leucine-stimulated mTOR activation in a TSC1/2 and Rheb-independent manner. (**A**), Serum-starved TSC1/2^+/+^ (WT) and TSC1/2^−/−^ (KO) MEF cells were pretreated with or without 50 µM for 60 min and then treated with or without 10 mM leucine (Leu) for 60 min. The phosphorylation and protein levels of S6K, 4E-BP1 and the protein levels of TSC1 and TSC2 in cell lysates were determined by Western blot with the indicated antibodies. (**B**), S6K phosphorylation shown in (**A**) was semi-quantified and normalized to S6K protein levels. (**C**), serum-starved C2C12 cells transiently expressing Rheb or Rheb^S16H^ were treated with or without 50 µM UA for 1 hour, followed with or without 10 mM leucine (Leu) for 60 min. The phosphorylation and protein levels of S6K, and the protein levels of FLAG-Rheb in cell lysates were determined by Western blot using indicated antibodies. (**D**), S6K phosphorylation in (**C**) was semi-quantified using the NIH Image J Program and normalized with S6K protein levels. Differences between groups were examined for statistical significance using ANOVA. Data are presented as mean ± S.E.M. from three independent experiments. *, P<0.05, **, P<0.01, and ***, p<0.001. N, no addition.

Branched-chain amino acids such as leucine have been found to activate mTOR complex 1 (mTORC1) via inhibition of TSC1/TSC2 [32], [33]. To determine whether UA inhibits leucine-stimulated mTOR signaling by targeting TSC1/2, we examined whether knockout of TSC1/2 affects the ability of UA to suppress mTORC1 signaling. Basal and leucine-stimulated S6K and 4E-BP1 phosphorylation is increased in TSC1/2 knockout MEFs compared to control MEFs (Figs. 2A and 2B). UA treatment inhibited leucine-stimulated phosphorylation of S6K and 4E-BP1 in not only control MEFs but also TSC1/2-null MEFs (Figs. 2A and 2B), suggesting that UA inhibits leucine-stimulated mTOR signaling by acting at a site downstream of TSC1/2 in the mTORC1 signaling pathway.

The small GTPase Rheb has been shown to be necessary for amino acid-dependent mTORC1 activation [16]. To determine the potential involvement of Rheb in UA-induced suppression of mTORC1 signaling, we examined S6K phosphorylation in C2C12 cells stably expressing wild-type Rheb and the constitutively active Rheb^S16H^. S6K phosphorylation was enhanced in cells overexpressing Rheb^WT^ and Rheb^S16H^ and the increase was augmented by treatment of the cells with leucine (Figs. 2C and 2D). While pre-treatment of the C2C12 cells with UA had little effect on Rheb overexpression-induced phosphorylation of S6K, it greatly inhibited the leucine-stimulated phosphorylation of S6K (Fig. 2C and 2D), suggesting that UA inhibits leucine-stimulated mTORC1 signaling via a Rheb activity-independent mechanism.

### UA-mediated Inhibition of the mTORC1 Signaling Pathway is Independent of the Interaction between mTOR and Raptor/Deptor

**Figure 3:**
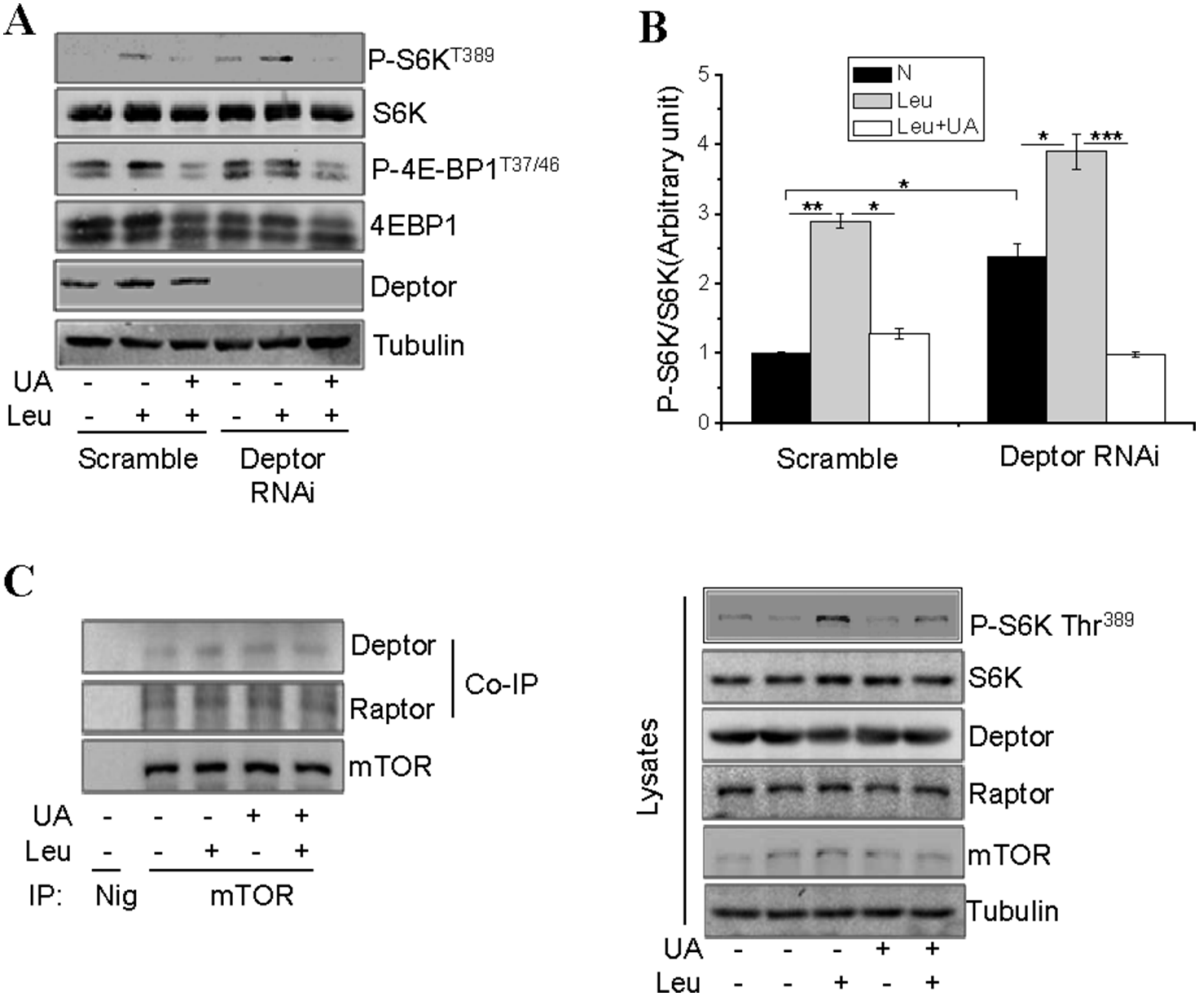
UA does not inhibit the associations between mTOR and Raptor or Deptor. (**A**), Deptor-suppressed and scrambled C2C12 cells were serum starved and pretreated with or without 50 µM UA for 60 min, followed with or without 10 mM leucine (Leu) for 60 min. The phosphorylation of S6K, 4EBP1 and the protein levels of S6K, 4EBP1, Deptor, and Tubulin were determined by Western blot using specific antibodies. (**B**), S6K phosphorylation in (**A**) was semi-quantified using the NIH Image J Program and normalized with S6K protein levels. (**C**), C2C12 cells were serum starved and pretreated with or without leucine (Leu) for 60 min, followed with or without 50 µM UA for 60 min. mTOR was immunoprecipitated from cell lysates using an anti-mTOR antibody and the co-immunoprecipitaed Raptor or Deptor was determined by Western blot. Differences between groups were examined for statistical significance using ANOVA. Data are presented as mean ± S.E.M. from three independent experiments. NIg, normal immunoglobulin. *, P<0.05, **, P<0.01, and ***, p<0.001. N, no addition.

To determine the potential involvement of the negative regulator Deptor in UA-induced inhibition of the mTORC1 signaling, we examined the effect of UA on leucine-stimulated S6K phosphorylation in C2C12 cells in which the expression levels of Deptor were suppressed by RNAi. As expected, mTORC1 signaling was increased in Deptor-suppressed cells, as demonstrated by increased basal S6K and 4EBP-1 phosphorylation (Figs. 3A and 3B). Leucine treatment further enhanced S6K phosphorylation in Deptor-suppressed cells and the stimulatory effect of leucine was reduced by UA treatment (Figs. 3A and 3B). Consistent with this, UA treatment had little effect on the interaction between mTOR and Deptor (Fig. 3C). Taken together, these results suggest that the interaction between Deptor and mTOR does not mediate the inhibitory effect of UA on leucine-stimulated mTOR activation. Since mTORC1 is activated by interaction with Raptor [34], we also examined the effect of UA on the association between mTOR and Raptor. We found that UA treatment had no significant effect on the association between mTOR and Raptor (Fig. 3C), suggesting that UA inhibits leucine-stimulated mTOR signaling by a distinct mechanism.

### UA Inhibits Leucine-stimulated mTORC1 Signaling by Disrupting mTOR Lysosomal Localization

**Figure 4:**
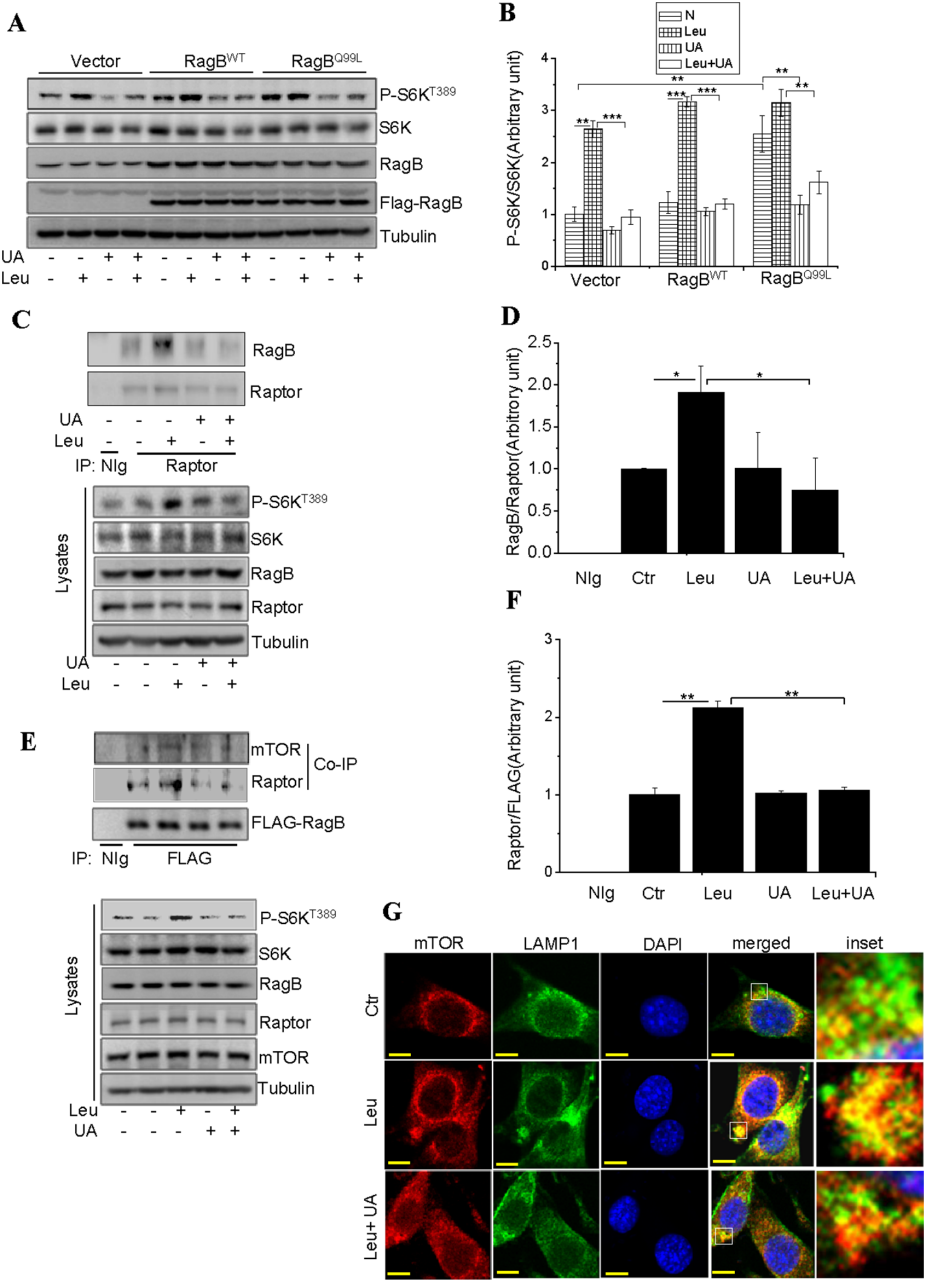
UA inhibits leucine-stimulated mTORC1 activity by disrupting mTOR lysosomal localization. (**A**), C2C12 cells transiently expressing control vector, RagB or RagB^GTP^ were serum starved and treated with or without 50 µM UA for 60 min, followed with or without 10 mM leucine (Leu) for 60 min. The phosphorylation and protein levels of S6K and the protein levels of RagB, FLAG, and Tubulin in cell lysates were determined by Western blot using specific antibodies as indicated. (**B**), S6K phosphorylation in (**A**) was semi-quantified using the NIH Image J Program and normalized to the levels of S6K in cells. (**C**), C2C12 cells transiently expressing RagB were serum starved and pretreated with or without with or without 50 µM UA for 60 min, followed with or without 10 mM leucine (Leu) for 60 min. Cells were then treated with the cross-linker chemical DSP and lysed. Raptor was immunoprecipitated from cell lysates and its associated proteins were detected by Western blot. (**D**), The cellular levels of RagB in (**C**) was semi-quantified using the NIH Image J Program and normalized to Raptor. (**E**), Serum-starved C2C12 cells transiently expressing RagB were pretreated with or without leucine (Leu) for 60 min and then with 50 µM UA for 60 min. Cells were then treated with DSP and lysed. FLAG-taged RagB was immunoprecipitaed from cell lysates and the associated proteins were determined by Western blot using antibodies to mTOR or Raptor. (**F**), the levels of Raptor in (**E**) was semi-quantified using the NIH Image J Program and normalized to FLAG-RagB. Differences between groups were examined for statistical significance using ANOVA. Data are presented as mean ± S.E.M. from three independent experiments. NIg, normal immunoglobulin. *, P<0.05, **, P<0.01, and ***, P<0.001. N, no addition. (**G**), serum-starved C2C12 cells were pretreated with or without 50 µM UA for 60 min, followed with or without 10 mM leucine (Leu) for 60 min. Cells were stained using antibodies specific to mTOR (red) and LAMP1 (green) and visualized by a confocal immunofluorescence microscope. Inset: Higher magnification to show colocalization (yellow) of mTOR and LAMP1. Scale bar, 10 µm.

The small GTPase Rag has been shown to be critical for leucine-stimulated mTORC1 activation [16], [35]. To determine if UA inhibits mTORC1 signaling by targeting Rag GTPase, wild-type RagB or the constitutively active RagB^Q99L^ were transiently expressed in C2C12 cells. Overexpression of RagB per se had no significant effect on S6K phosphorylation but potentiated leucine-stimulated S6K phosphorylation (Figs. 4A and 4B). Overexpression of RagB^Q99L^, on the other hand, significantly promoted basal S6K phosphorylation, which was not further enhanced by leucine treatment (Figs. 4A and 4B). Treatment of the cells with UA inhibited leucine-stimulated mTORC1 signaling in cells overexpressing either RagB or RagB^Q99L^ (Figs. 4A and 4B), suggesting that UA acts at RagB or at a site downstream of RagB. To further test this, we examined the effect of UA on the association between RagB and Raptor, the later has been shown to mediate the interaction between RagB and mTORC1 [16]. Co-IP experiments showed that leucine treatment significantly stimulated the interaction between RagB and Raptor (Figs. 4C–4D). Similar result was obtained by reciprocal Co-IP experiments (Fig. 4E and 4F). However, the leucine-stimulated association of RagB with Raptor and mTOR was inhibited by UA (Figs. 4C–4F).

To further elucidate the mechanism by which UA inhibits mTORC1 signaling, we examined RagB-mediated recruitment of mTORC1 to lysosome, which is critical for leucine-stimulated mTORC1 activation [18]. Confocal immunofluorescence experiments showed that treatment of the C2C12 cells with leucine promoted the co-localization of mTOR with the lysosomal marker LAMP1 (Fig. 4G). The co-localization of mTOR with LAMP1 was greatly suppressed by pre-treating the cells with UA (Fig. 4G), suggesting a mechanism by which UA inhibited leucine-stimulated activation of the mTORC1 signaling pathway.

## Discussion

UA has been shown to exert many beneficial effects such as anti-diabetes [36], anti-obesity [37], and anti-cancer [38]. Several recent studies show that UA exerts its anti-tumor role through inhibition of the mTOR signaling pathway [39], suggesting a potential mechanism underlying the beneficial effects of UA. However, how UA inhibits mTORC1 signaling pathway remains unknown.

C2C12 myotubes possess morphological, biochemical and metabolic properties of the isolated skeletal muscle [40], [41] and are widely used for studying muscle cell function and physiology. Here we show that UA inhibits insulin- and leucine-stimulated mTORC1 signaling in C2C12 cells (Figs. 1A–1D). While UA is able to inhibit insulin-stimulated mTORC1 signaling by negative regulation of Akt, the inhibition of leucine-induced mTORC1 activation by UA appears to be mediated by an Akt-independent novel mechanism. As shown in figure 3, the UA-mediated inhibition of the mTORC1 signaling pathway is independent of the cellular levels of Raptor and Deptor, two key regulators of the mTORC1 signaling pathway [11], [34], [42]. On the other hand, treating C2C12 myoblasts with UA suppressed the interaction between Raptor and RagB (Figs. 4C–4F), and inhibited the recruitment of mTOR onto the lysosomal surface (Fig. 4G), a key step in the amino acid**–**induced activation of mTORC1 [16], [43]. Although UA has little effect on the association between mTOR and Raptor, the prevention of mTOR or Raptor from binding to RagB (Figs. 4C–4F) may provide a mechanism for UA to suppress the recruitment of mTOR to the lysosomal surface for activation.

How UA disrupts the binding of RagB to Raptor remains unknown. One possibility may be that UA directly interacts with RagB or mTORC1 components, leading to a conformational change of these proteins and thus impairs their interaction. Since the interaction between mTOR with the Rag proteins is critical for mTOR localization to the lysosome in response to amino acid stimulation [44], disruption the interaction between RagB and mTORC1 components could be the mechanism by which UA suppresses leucine-stimulated mTOR localization to the lysosome. Alternatively, UA may inhibit the conversion of GDP-bound inactive form of RagB to the GTP-bound active forms, thus preventing RagB interaction with mTOR. Future studies are needed to clarify these possibilities.

In summary, we have demonstrated for the first time that UA inhibits leucine-stimulated mTOR signaling in C2C12 cells. In addition, we show that UA inhibits mTORC1 signaling by disrupting the interaction between RagB-mTORC1. As aberrant activation of the mTORC1 signaling pathway is associated with impaired energy homeostasis and increased incidents of human tumors/cancers [45], [46], identification of small molecular inhibitors of this signaling pathway and elucidating their mechanisms of action are critical for the prevention and treatment of these diseases.
